# Unfractionated Heparin Improves the Intestinal Microcirculation in a Canine Septic Shock Model

**DOI:** 10.1155/2021/9985397

**Published:** 2021-06-23

**Authors:** Heng Zhang, Yini Sun, Xin An, Xiaochun Ma

**Affiliations:** ^1^Department of Neurosurgery, The First Affiliated Hospital of China Medical University, Shenyang, China; ^2^Department of Critical Care Medicine, The First Affiliated Hospital of China Medical University, Shenyang, China

## Abstract

**Background:**

Alterations of microcirculation are associated with organ hypoperfusion and high mortality in septic shock. This study is aimed at investigating the effects of unfractionated heparin (UFH) on intestinal microcirculatory perfusion and systemic circulation in a septic shock model.

**Methods:**

Twenty-four beagle dogs were randomly allocated into four groups: (a) sham group: healthy controls, (b) shock group: septic shock induced by *Escherichia coli*, (c) basic therapy group: septic shock animals treated with antibiotics and 10 ml/kg/h saline, and (d) heparin group: septic shock animals treated with basic therapy plus UFH. Hemodynamic variables were measured within 24 h after *E. coli* administration. The intestinal microcirculation was simultaneously investigated with a sidestream dark-field imaging technique. Additionally, the function of vital organs was evaluated at 12 h postadministration (T12).

**Results:**

*E. coli* induced a progressive septic shock in which the mean arterial pressure (MAP) decreased and lactate levels sharply increased, accompanied by deteriorated microvessel perfusion. While basic therapy partially improved the microvascular flow index and the perfused microvessel density in the jejunal villi, UFH significantly restored major microcirculation variables at T12. Physiological variables, including MAP, urine output, and lactate levels, were improved by UFH, whereas some hemodynamic indices were not affected by UFH. With respect to organ function, UFH increased the platelet count and decreased the creatinine level.

**Conclusions:**

UFH improves microcirculatory perfusion of the small intestine independently of the changes in systemic hemodynamic variables in a canine model of septic shock, thereby improving coagulation and renal function.

## 1. Introduction

Sepsis is a life-threatening organ dysfunction caused by a dysregulated host response to infection and is associated with high mortality [1]. Fluid resuscitation has been demonstrated to improve hemodynamic variables and stabilize the balance between oxygen supply and consumption, which leads to decreased mortality [2]. However, if antibiotics and fluid resuscitation treatment fails, no additional approved effective therapeutics are available [3]. Increasing evidence shows that intraorgan blood flow defects caused by microcirculatory dysfunction contribute to the development of organ failure and poor prognosis in septic shock patients [4, 5].

Tissue perfusion insufficiency, including systemic or regional alteration, plays a crucial role in organ dysfunction. Research has proven that organ dysfunction may persist, despite apparent restoration of systemic macrohemodynamics [6]. Therefore, restoring microcirculation may be the therapeutic target in septic shock. The microcirculatory system is composed of arterioles, venules, and capillaries, where substrate exchange takes place [7]. Microcirculation disorders could be caused by the endothelial injury induced by proinflammatory cytokines, microthrombosis, and glycocalyx degradation at the early stage of septic shock [8]. Microcirculation dysfunction acts as the “motor” of driving pathogenic changes in sepsis [9]. Recent studies have indicated that intestinal microcirculatory dysfunction can be observed at the early stage of sepsis and septic shock [10] and in a hemorrhagic shock model [11]. A dissociation between sublingual and intestinal microcirculation has been found in septic patients [12]. Experimental research on large animals has shown that fluid resuscitation normalizes sublingual microcirculation, while intestinal villi remain hypoperfused [13]. Microcirculation in the intestinal villi mucosa better represents the perfusion of vital internal organs than sublingual detection. Therefore, the intestine is considered to be a priority site to investigate sepsis-induced microcirculatory dysfunction.

Unfractionated heparin (UFH) is an acidic glycosaminoglycan mainly generated by mast cells and basophils. It acts as an anticoagulant, preventing thrombosis by increasing the affinity between antithrombin III and proteases, thereby accelerating the inactivation of proteases. Except for anticoagulant and anti-inflammatory properties, UFH can efficiently restore the morphology and function of vascular endothelial cells, prevent glycocalyx shedding, and improve organ perfusion [14]. Our previous studies have demonstrated that UFH inhibits overinflammatory responses and prevents endothelial deterioration, which result in improved organ function and survival in sepsis [15–17]. Recent studies have revealed that UFH improves the 28-day mortality in septic patients by reducing disseminated intravascular coagulation without increasing the risk of bleeding [18, 19]. Therefore, UFH may improve organ perfusion and microcirculation during sepsis.

Here, we report that *Escherichia coli*-induced deterioration of jejunal mucosa microcirculation was accompanied by hemodynamic disorder and organ dysfunction in a canine septic shock model. While UFH slightly improved some physiological variables, such as the mean arterial pressure (MAP), urine output (UO), and the lactate clearance rate, it (i) significantly improved jejunal microcirculatory dysfunction and (ii) accompanies the normalization of the coagulation and renal function in a canine septic shock model.

## 2. Materials and Methods

### 2.1. Animals

Twenty-four adult beagle dogs (12 males and 12 females), weighing 13.31 ± 1.65 kg, were purchased from the Laboratory Animal Center of Dalian Medical University (animal qualification number, SCXX [Liao] 2008-0002). All of the animals had free access to standard dog food and water throughout the experiments. They were kept on a strict day/night light cycle at a room temperature of 22 ± 1°C and a relative humidity of 30%-60% for a minimum of 10 days before surgery. All of the experiments were performed following the *Guide for the Care and Use of Laboratory Animals* (8^th^ edition, 2011) and were approved by the Ethics Committee for Animal Research of China Medical University (protocol number L2011001).

### 2.2. Bacterial Culture

The *Escherichia coli* (*E. coli*, serotype O79) bacteria strain was provided by the Department of Laboratory Medicine of the First Hospital of China Medical University [20]. This strain is sensitive to ceftriaxone sodium. The bacteria were cultured on the sheep blood agar plates. The plates were incubated at 37°C for 16 h in 5% CO_2_, and colony counts were determined. The McBurney turbidity (Mc-fold) of the bacterial suspension was measured using spectrophotometry. According to the preliminary experiment, a bacterial amount of 3.5 × 10^8^ colony-forming units (cfu)/kg was administrated to each animal. The bacterial suspension was mixed with 20 ml sterile saline before injection.

### 2.3. Surgical Preparation

The animals underwent surgery under sterile conditions. Anesthesia was induced by intramuscular injections of pentobarbital (25 mg/kg). Sedation and analgesia were maintained through the continuous intravenous infusion of pentobarbital (2-3 mg/kg/h) combined with fentanyl (0.3-0.4 *μ*g/kg/h) until the animals were euthanized. Intravascular catheters were placed in the femoral arteries and external jugular veins. The animals were orally intubated with a 7.5 ID tracheal tube for mechanical ventilation (Servo Ventilator 900C, Siemens-Elema; ventilation setting: pressure control; Pi: 12-16 cm H_2_O; respiratory rate: 12-14/min; FiO_2_: 23%-25%), in order to maintain SpO_2_ > 95%, pO_2_ > 80 mmHg, and pCO_2_ = 35 ± 5 mmHg. A heating pad was used to maintain the core body temperature at 36.5 ± 1.0°C. Cystostomy was performed with a urethral catheter for adults (14F) fixed to the abdominal wall through a paramedian incision. Additionally, a gastric tube was orally placed for gastrointestinal decompression. Jejunostomy was performed over a length of 15-20 cm from the Treitz ligament, and the orificium fistulae were fixed at the lateral abdomen for intestinal microcirculation observation.

### 2.4. Experimental Protocol

The 24 canines were randomly allocated into four groups. (a) Sham group: intravascular catheters were placed and microcirculation of intestinal villi was monitored. (b) Shock group: *E. coli* suspension (3.5 × 10^8^ cfu/kg) was injected intravenously into the animals at 3 ml/kg/h. When the MAP decreased to 80% of the baseline, the timepoint (approximately 1 h postinjection) was designated as the establishment of the septic shock model [21]. (c) Basic therapy group: when the septic shock model was established, the animals were intravenously administered with 0.9% saline at 10 ml/kg/h for fluid resuscitation. And from 1 h postinjection, ceftriaxone was administrated at 37.5 mg/kg every 12 h. (d) Heparin group: after the establishment of the model, the animals were given a continuous injection of 40 IU/kg/h UFH [22] (No.1 Biochemistry & Pharmaceutical Co., Shanghai, China) and 10 ml/kg/h saline. Antibiotics (ceftriaxone, 37.5 mg/kg) were administered simultaneously. The animals were observed for 24 h or until death. The total fluid input and output were calculated, and the blood withdrawn was less than 10% of the estimated total blood volume. Urine volume was recorded hourly. At the end of the experiment, the animals were euthanized with an overdose of potassium chloride.

### 2.5. Microcirculation Measurements

The microcirculation of the jejunal villi was visualized with a sidestream dark-field (SDF) illumination imaging system (Microscan, Amsterdam, the Netherlands). The probe of the SDF was inserted to the same depth (5-7 cm) from the fistula of the abdominal wall. We collected five high-quality steady images of at least 20 s in different regions of the jejunum using a hand-held SDF device according to recommended techniques for video acquisition [23]. All the digital images were stored and automatically analyzed by AVA 3.0® software (Academic Medical Center, University of Amsterdam, the Netherlands). Special care was taken to avoid putting pressure on the microvessels. We recorded sublingual videos at T0 (before *E. coli* injection), T1, T2, T3, T4, T6, T8, T12, T18, and T24. The internal surface of the intestine was washed with saline at 37°C to maintain the same temperature conditions for microcirculation observation. The vascular video analysis was conducted manually and blindly by two independent investigators (Z. Heng and A. Xin). We used the following parameters to assess intestinal microcirculation, as suggested by Schiffer et al. [23]: total vessel density (TVD, mm/mm^2^), perfused vascular density (PVD, mm/mm^2^), proportion of perfused vessels (PPV, %), and microvascular flow index (MFI). The TVD was calculated as the total length of vessels divided by the total area of the image. The PVD was calculated as the length of perfused vessels divided by the total area of the image. The PPV was calculated as follows: PPV = (total number of vessels − numbers of vessels without flow)/total number of vessels. The MFI was evaluated as described by Schiffer et al. [23]. The image was divided into four quadrants, and the predominant flow type of the small vessels was assessed in each quadrant, where 0 indicates the absence of flow, 1 indicates intermittent flow, 2 indicates sluggish flow, and 3 indicates normal flow. The MFI score was calculated as the average of the values of the four quadrants. All the variables were given as the average value of five sites.

### 2.6. Physiological and Hemodynamic Variables and Organ Function Measurements

The timepoint of *E. coli* administration was designated as T0. *E. coli* suspension (3.5 × 10^8^ cfu/kg) was continuously injected from T0 to T2 *via* the external jugular vein. Blood samples were taken, and hemodynamic measurements were performed at T0, T1, T2, T3, T4, T6, T8, T12, T18, and T24. Hemodynamic variables were monitored by a pulse indicator continuous cardiac output (PICCO) system (PICCOPlus PC 8100, PULSION Medical Systems AG, Munich, Germany) through the catheter in the right femoral artery. MAP, heart rate (HR), cardiac index (CI), systemic vascular resistance index (SVRI), and stroke volume variation (SVV) were measured at designated timepoints. The PICCO system was calibrated by injecting 5 ml saline at 0°C three times via the external jugular vein. Samples for blood gas analysis were collected in heparinized 1 ml syringes. The variables representing organ function, including the platelet count, oxygenation index, hepatic alanine transaminase (ALT), and creatinine (Cr) levels, were measured at T12.

### 2.7. Statistical Analysis

Normally distributed data are expressed as the mean ± standard error of the mean. Dynamic data were analyzed over time and between groups by two-way analysis of variance (ANOVA) followed by Dunnett's multiple comparisons test. For a single timepoint, the four groups were compared by one-way ANOVA, followed by Dunnett's test or the LSD multiple comparisons test. Nonnormally distributed data are expressed as medians (interquartile range) and were analyzed with the Kruskal-Wallis test, followed by post hoc Mann-Whitney *U* tests with adjustment for multiple comparisons. Two-tailed *P* values < 0.05 were considered statistically significant. All statistical analyses were conducted using SPSS 22.0 software (IBM SPSS Statistics, Chicago, IL, USA) and GraphPad Prism 7.0 software (San Diego, CA).

## 3. Results

### 3.1. The Establishment of the Septic Shock Model

**Figure 1:**
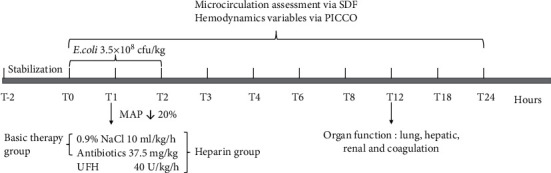
Study design. All animals were stabilized for 2 hours after surgical procedures, and 3.5 × 10^8^ cfu/kg *E. coli* was continuously injected from T0 to T2. Hemodynamics and microcirculation measurements were taken over a 24 h period: at baseline (T0), T1, T2, T3, T4, T6, T8, T12, T18, and T24.

Twenty-four age- and gender-matched beagle dogs were randomly allocated into four groups. Each group included three female and three male animals. The MAP sharply decreased after *E. coli* infusion. When the MAP decreased to 80% of the baseline (approximately at T1), this was defined as the establishment of the septic shock model. From this moment, therapy was administrated into the animals (Figure 1). Two of the six animals in the basic therapy group and four of the six animals in the heparin group survived until T24. The mean survival time was 11.9 ± 1.5 h in the shock group, 20.8 ± 1.7 h in the basic therapy group, and 22.8 ± 1.2 h in the heparin group. No major bleeding or other side effects were observed during the entire experiment. Additionally, no difference in the volume of intravenous fluid administrated for resuscitation was observed between the basic therapy and heparin groups (10.2 ± 1.2 vs. 10.3 ± 1.4 ml/kg/h, *P* > 0.05).

### 3.2. The Effects of UFH on the Microcirculation of Jejunal Villi in Septic Shock

**Figure 2:**
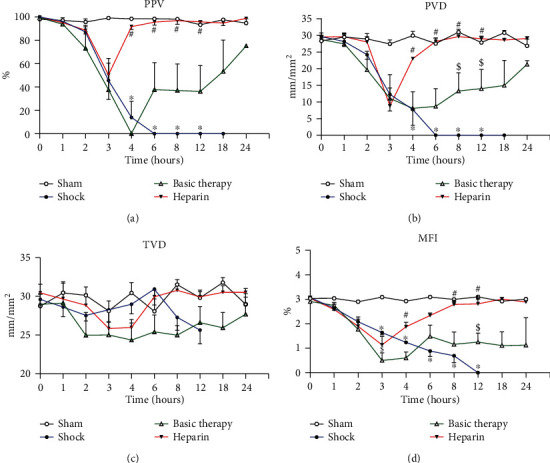
The dynamic changes of microcirculation in septic shock. The microcirculatory parameters including PPV, PVD, TVD, and MFI were monitored over 24 h and compared in the four groups. ^∗^*P* < 0.05: shock vs. sham group; ^#^*P* < 0.05: heparin vs. basic therapy group; ^$^*P* < 0.05: shock vs. basic therapy group.

The changes in the microcirculation of jejunal villi were visualized via SDF imaging technique. *E. coli* infusion aggravated major microcirculatory variables, including the PPV, PVD, and MFI. The PPV sharply and continuously declined until the end of the study in the shock group (sham vs. shock group: *P* < 0.0001 for T4, T6, T8, and T12). PPV decreased to zero at T4 and then slowly increased in the basic therapy group. Compared with the basic therapy group, UFH normalized the PPV from T3 (Figure 2(a)). Similarly, the PVD declined sharply postinjection in the shock group compared to the sham group (*P* < 0.001 for T4, T6, T8, and T12; Figure 2(b)). Basic therapy partially increased the PVD compared to the shock group (T8, *P* = 0.011; T12, *P* = 0.014; Figure 2(b)). Of note, the PVD was recovered from T4 in the heparin group and increased to the same level as in the sham animals from T6 to T24. No significant differences in changes in the TVD were noted among the four groups (Figure 2(c)). Furthermore, the MFI significantly decreased from T3 to T12 in the shock group (*P* < 0.001). The MFI did not significantly increase in the basic therapy group. However, the MFI significantly increased from T4 after a transient decline in the heparin group (*P* < 0.001 for T4, T8, and T12; Figure 2(d)).

**Figure 3:**
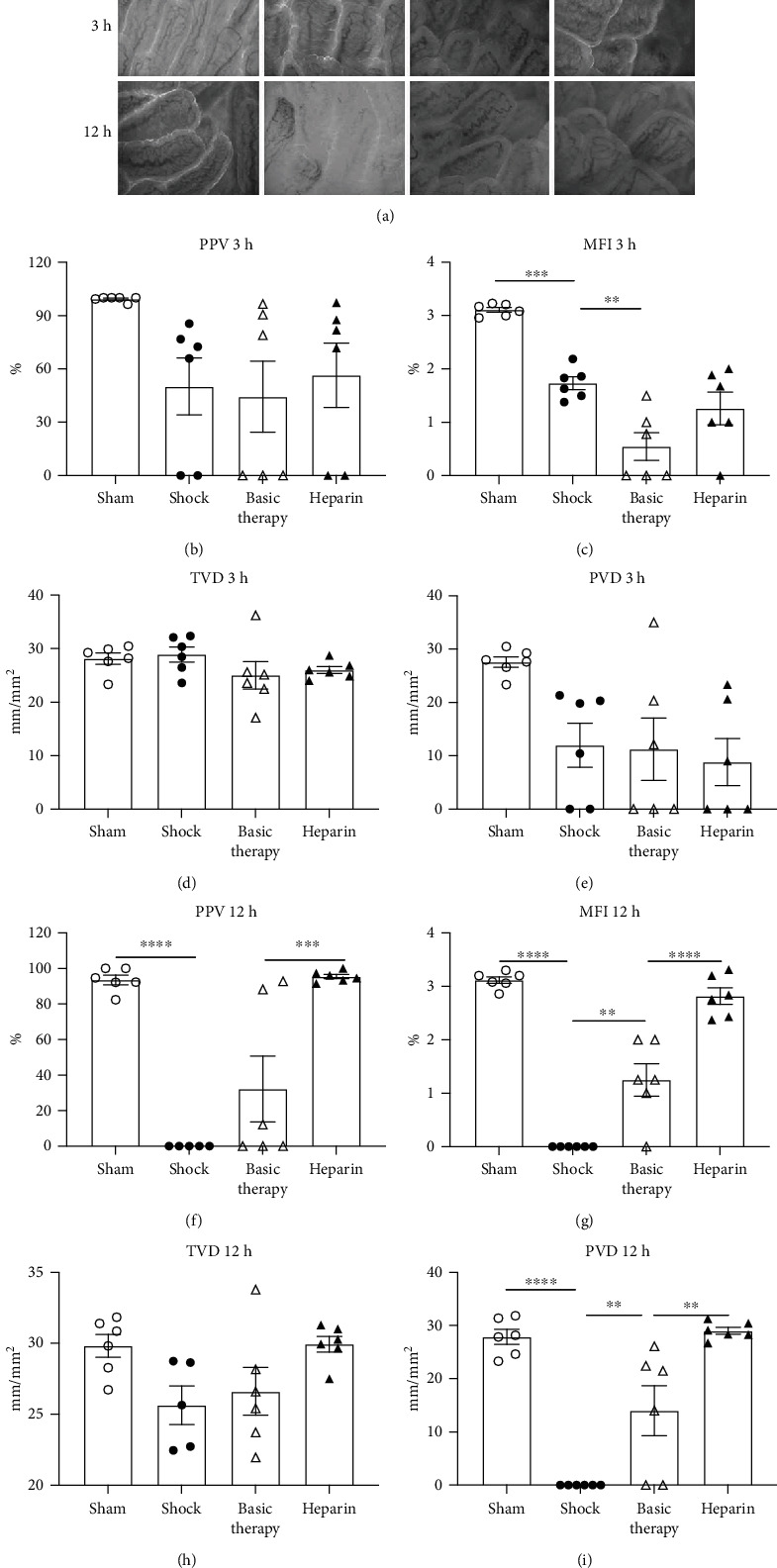
Representative images and parameters of microcirculation in the jejunal villi at T3 and T12. (a) Representative images of jejunal villus mucosa at T3 and T12. (b–e) Microcirculation parameters PPV, MFI, TVD, and PVD of jejunal villi at T3. (f–i) The PPV, MFI, TVD, and PVD of jejunal villi at T12. ^∗∗^*P* < 0.01; ^∗∗∗^*P* < 0.001; ^∗∗∗∗^*P* < 0.0001.

Representative microvascular images of jejunal villi at T3 and T12 in the four groups are shown in Figure 3(a). Neither PVD nor MFI was improved by basic therapy or the addition of UFH at T3 (Figures 3(b)–3(e)). However, while improvements in PPV, MFI, and PVD were not detected in the shock group, basic therapy increased the MFI and PVD at T12 (Figures 3(f)–3(i)). Compared to the basic therapy group, UFH further significantly increased the PPV (basic therapy vs. heparin: 32 ± 18.5% vs. 95 ± 1.2%, *P* = 0.0009; Figure 3(f)) and MFI (*P* < 0.0001; Figure 3(g)) at T12. Although the TVD was not significantly improved by either basic therapy or UFH (Figure 3(h)), the PVD was increased by UFH compared to the basic therapy group (14 ± 4.7 vs. 29 ± 0.6 mm/mm^2^, *P* = 0.0019; Figure 3(i)) at T12.

### 3.3. The Effects of UFH on Physiological Variables in Septic Shock

**Figure 4:**
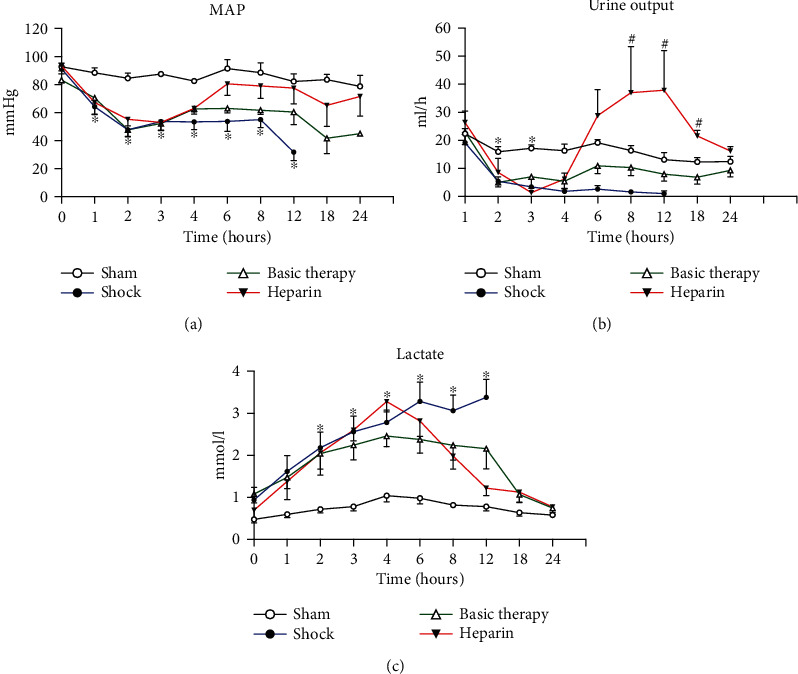
The dynamic characterization of basic physiology index in septic shock. MAP, UO, and the blood lactate level were compared along time among the four groups. ^∗^*P* < 0.05: shock group vs. sham group; ^#^*P* < 0.05: heparin group vs. basic therapy group.

At T1, MAP was decreased by 15%-27% in all groups except for the sham group. In the shock group, the MAP sharply declined during the first 2 h, slowly decreased until T8, and dramatically dropped after T8. In the basic therapy and heparin groups, the MAP increased from T4. Compared with the basic therapy, the addition of UFH tended to increase the MAP from T4 to the end of the study (Figure 4(a)). UO significantly decreased following *E. coli* administration (*P* < 0.05). UO tended to increase from T4 in the basic therapy group. Furthermore, UFH dramatically improved UO from T4 to T24 compared to the basic therapy group (*P* < 0.05; Figure 4(b)). *E. coli* produced a sustained increase in arterial lactate concentration in the first 4 h (*P* < 0.05). While the lactate level consistently rose in the shock animals, it tended to decrease from T12 in the basic therapy group and from T6 in the heparin group (Figure 4(c)).

**Figure 5:**
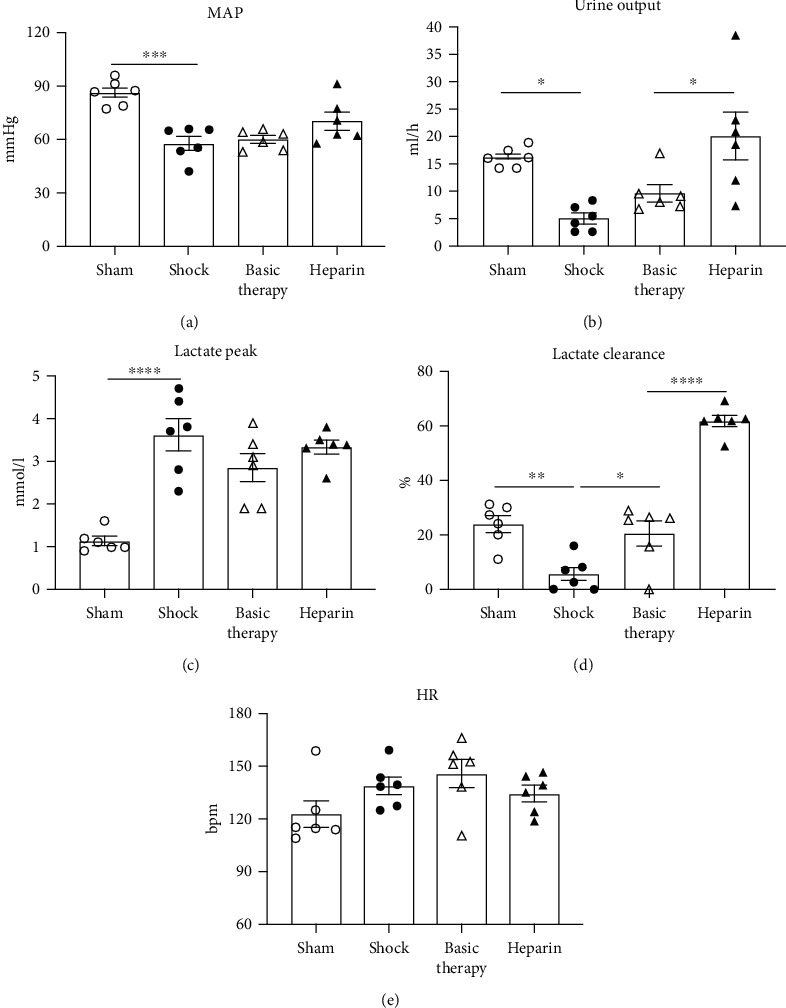
The effect of UFH on basic physiology index in septic shock. The parameters were represented as the average values of MAP, UO, the lactate level, and HR from T0 to T24. ^∗^*P* < 0.05; ^∗∗^*P* < 0.01; ^∗∗∗^*P* < 0.001; ^∗∗∗∗^*P* < 0.0001.

The mean values of MAP, UO, lactate, and HR were calculated from T0 to T24 to better compare the differences among the four groups. The MAP and UO decreased across 24 h in the shock group relative to the sham group. However, UFH significantly enhanced the mean UO (Figures 5(a) and 5(b)). To further assess lactate levels, we analyzed the peak value of arterial lactate levels and the lactate clearance rate during 6 h following the peak. Compared with the sham animals, the lactate peak level was significantly higher in the shock group (Figure 5(c)). Furthermore, the lactate clearance rate was significantly lower in the shock group relative to the sham group (*P* = 0.003), and the lactate clearance rate increased in the basic therapy group (Figure 5(d)). Importantly, UFH significantly increased the lactate clearance rate (*P* < 0.0001, Figure 5(d)). The HR did not differ among the four groups (Figure 5(e)).

### 3.4. The Effects of UFH on Hemodynamic Variables via PICCO in Septic Shock

**Figure 6:**
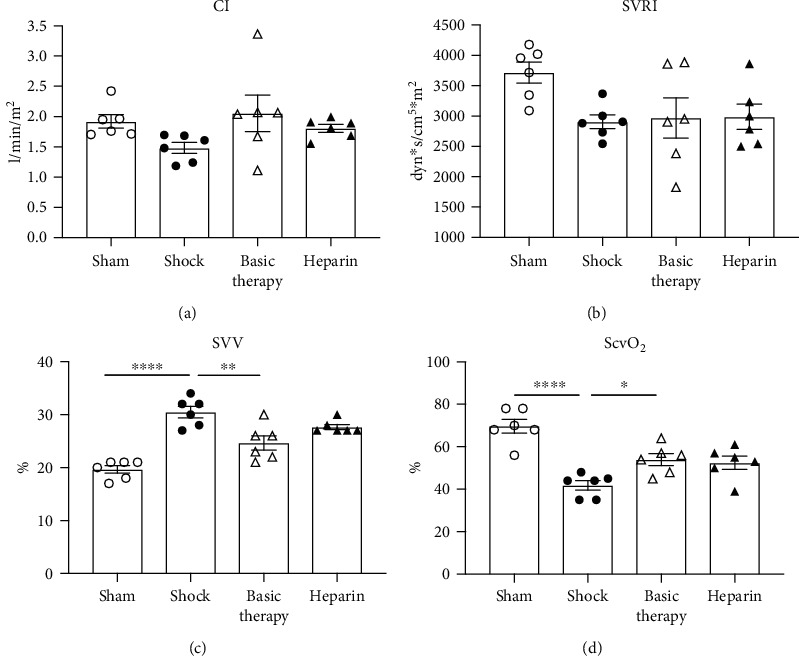
The effect of UFH on systemic hemodynamic variables in septic shock. (a–c) Cardiac index (CI), systemic vascular resistance index (SVRI), and stroke volume variation (SVV) were acquired from PICCO. (d) Central venous oxygen saturation (ScvO_2_) was obtained from blood gas analysis. ^∗^*P* < 0.05; ^∗∗^*P* < 0.01; ^∗∗∗∗^*P* < 0.0001.

To evaluate the effects of UFH on systemic macrohemodynamics, we explored the differences in hemodynamic variables among the four groups via a PICCO system. The average value of the CI across the 24 h did not significantly differ among the four groups (Figure 6(a)). The SVRI, an index to assess the cardiac afterload, tended to decrease in the shock group relative to the sham group. Basic therapy or heparin did not improve the SVRI (Figure 6(b)). To assess the cardiac preload, we also evaluated the mean value of the SVV across the study period. Compared with the sham animals, *E. coli* infusion induced a significant increase in SVV in the shock group (*P* < 0.0001; Figure 6(c)). The SVV dramatically declined in the basic therapy group compared with the shock group, whereas heparin did not affect the SVV (Figure 6(c)).

To further assess oxygen delivery, we measured the central venous oxygen saturation (ScvO_2_) across 24 h in the four groups. ScvO_2_ in the shock group showed a sharp decrease relative to the sham animals. Additionally, UFH improved ScvO_2_ (Figure 6(d)).

### 3.5. The Effects of UFH on Organ Function in the Septic Shock Model

**Figure 7:**
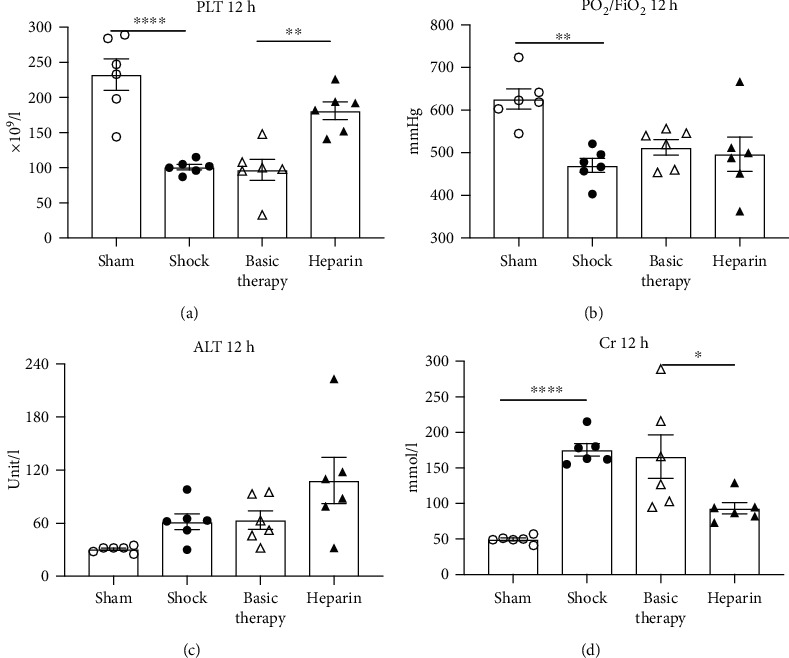
The effect of UFH on organ function in septic shock. (a) Platelet counts were achieved from complete blood count. (b) PO_2_/FiO_2_ was calculated from the arterial gas analysis results. (c, d) The alanine aminotransferase (ALT) and creatinine (Cr) levels in the blood were collected from biochemical analysis at T12. ^∗^*P* < 0.05; ^∗∗^*P* < 0.01; ^∗∗∗∗^*P* < 0.0001.

Due to the intimate relationship of microcirculation and organ function, we next investigated the effect of UFH on coagulation, respiratory, hepatic, and renal functions in the septic shock model. While the platelet counts were reduced sharply in the shock group compared with the sham animals, UFH significantly increased platelet counts relative to the basic therapy group at T12 (Figure 7(a)). Additionally, the PaO_2_/FiO_2_ ratio progressively decreased in the shock group, whereas either basic therapy or UFH did not improve the oxygenation index (Figure 7(b)). No differences in ALT levels were noted among the four groups (Figure 7(c)). Importantly, renal function deteriorated since the blood Cr levels dramatically increased at T12, indicating that the renal function had deteriorated. While basic therapy had no effect on the Cr levels following *E. coli* administration, the addition of UFH significantly decreased the Cr levels (*P* = 0.016; Figure 7(d)).

## 4. Discussion

We demonstrate that the UFH plus basic therapy (fluid+antibiotics) strategy effectively improved the microcirculation of the jejunum, resulting in renal and coagulation functional recovery, in an *E. coli*-induced canine septic shock model. Notwithstanding, UFH tended to increase the MAP, UO, and the blood lactate clearance rate relative to the basic therapy group. These data suggest that UFH exerts protective effects on intestinal microcirculation, improving organ perfusion and lengthening the period of survival in septic shock.

Here, we demonstrated that UFH ameliorated intestinal microcirculation by enhancing the PVD and microvessel flow following septic shock. First, UFH is widely used as an anticoagulant to prevent deep vein thrombosis in the clinic. Further, it has been demonstrated that UFH improves disseminated intravascular coagulation-associated microvascular thrombosis in sepsis [18]. Besides its anticoagulant properties, UFH has been reported to exert other clinical biological effects in the setting of sepsis [14]. Our previous studies revealed that UFH significantly ameliorates inflammatory responses and pulmonary microvascular endothelial barrier dysfunction, leading to increased survival in a lipopolysaccharide-induced sepsis model [17, 24]. Additionally, we previously demonstrated that UFH prevents endothelial glycocalyx shedding in a canine model of septic shock [15]. In previous studies, we also demonstrated that UFH attenuates intestinal injury by inhibiting heparanase and neutralizing histone-mediated cytotoxicity in a cecal ligation puncture rodent model [25, 26]. In the current study, we found that UFH significantly increased lactate clearance. Arterial lactate is considered a crucial index of organ perfusion and oxygen delivery, predicting the outcome of septic shock [1]. All these beneficial effects of UFH potentially contribute to improving microcirculation disorders and organ perfusion in septic shock.

Apart from increasing intestinal microvascular perfusion, UFH tended to improve renal function, as demonstrated by an increase in UO and a decrease in blood Cr concentration. Although we could not detect renal microvascular flow via SDF, this implies that UFH may exert beneficial effects on improving renal function in septic shock. The optimal dosage, duration, and safety of UFH treatment in sepsis remain controversial. In the present study, the dosage of UFH was determined on the basis of our preliminary experiments and by referring to the study by Schiffer et al. in an ovine endotoxin shock model [22]. Our findings are consistent with Schiffer et al.'s study in which UFH prevents endotoxin-induced coagulation disorders and markedly improved the survival rate [22]. During the study procedure, no hemorrhagic events were observed, which implies that the use of UFH is safe. In clinical practice, the efficacy and safety of UFH have been systemically analyzed [18]. Nevertheless, the optimal dosage of UFH for sepsis treatment remains to be further evaluated.

While UFH significantly improved sepsis-induced microcirculatory dysfunction, some hemodynamic variables were slightly affected by either basic therapy or UFH. A possible explanation for this observation is that young beagle dogs suffer from arrhythmia and tachycardia. HR increased upon septic shock and even more after fluid resuscitation. PICCO variables, such as CI, may be affected by tachycardia. It is noteworthy that the observed changes in macrocirculation were not parallel with changes in microcirculation in the septic shock model. Our results are consistent with previous experimental and clinical observations, which suggest that sepsis-related microcirculatory alteration is independent of systemic variables [27, 28]. Najakima et al. also reported endotoxin-induced microvascular blood flow alterations independent of changes in blood pressure [29].

We used fluid resuscitation and antibiotics as the standard treatment strategy. The PVD and MFI were significantly increased by basic therapy, compared with the shock group, suggesting the effectiveness of fluid resuscitation and antibiotics. However, it was also found that the increased fluid volume did not improve the hemodynamic parameters. In contrast, tissue edema and increased effusion in the lung were observed upon autopsy of animals. Consistent with previous studies, we confirmed that excessive fluid resuscitation and an inadequate response to fluid resuscitation led to tissue edema and increased mortality [4]. The additional infusion of UFH efficiently reversed the deteriorated microcirculation, which may further improve vital organ perfusion and the prognosis of septic shock.

The present study has several limitations. First, an analysis bias of the MFI cannot be ruled out as we have added undetected vessel segments and deleted vague vessels during SDF video analysis. However, this method has proven feasible and valuable in animal studies of cardiopulmonary resuscitation and sepsis. Second, alterations in microcirculation of other organs should be simultaneously monitored. Nonetheless, SDF can be applied to the microvascular system of superficial tissue, such as intestinal mucosa or sublingual mucosa. It is not suitable for other solid organs. Third, we did not investigate the mechanisms underlying the beneficial effects of UFH on microcirculation, which need to be elucidated in future studies.

## 5. Conclusion

The present study revealed that UFH could improve microcirculatory dysfunction independently of hemodynamic variables in a large animal model of septic shock. Our findings firstly reported that the intestinal microcirculation was improved significantly by the administration of UFH in a large animal model of septic shock, which imitates clinical septic shock in humans. Further, the result indicates that UFH improves coagulation and renal function. Taken together, we demonstrate that UFH could be a promising therapeutic candidate for the clinical treatment of Gram-negative sepsis.

## Data Availability

The datasets used and analyzed during the current study are available from the corresponding author on reasonable request.

## Abbreviations

UFH: Unfractionated heparin
PICCO: Pulse indicator continuous cardiac output
SDF: Sidestream dark-field
MFI: Microvascular flow index
PVD: Perfused microvessel density
PPV: Proportion of perfused microvessels
MAP: Mean arterial pressure
HR: Heart rate
CI: Cardiac index
SVRI: Systemic vascular resistance index
Lac: Lactate
ScvO_2_: Central venous oxygen saturation
SVV: Stroke volume variation
ALT: Alanine transaminase
Cr: Creatinine
UO: Urine output.
